# Estimated Association Between Cytokines and the Progression to Diabetes: 10-year Follow-Up From a Community-Based Cohort

**DOI:** 10.1210/clinem/dgz171

**Published:** 2019-11-06

**Authors:** Nam H Cho, Eu Jeong Ku, Kyoung Yeon Jung, Tae Jung Oh, Soo Heon Kwak, Jae Hoon Moon, Kyong Soo Park, Hak C Jang, Yoon Ji Kim, Sung Hee Choi

**Affiliations:** 1 Department of Preventive Medicine, Ajou University School of Medicine, Suwon, Republic of Korea; 2 Department of Internal Medicine, Chungbuk National University Hospital and Chungbuk National University College of Medicine, Cheongju, Republic of Korea; 3 Department of Internal Medicine, Nowon Eulji Medical Center, Eulji Hospital, Seoul, Republic of Korea; 4 Department of Internal Medicine, Seoul National University College of Medicine, Seoul, Republic of Korea; 5 Department of Internal Medicine, Seoul National University Bundang Hospital, Seongnam, Republic of Korea; 6 Department of Internal Medicine, Mediplex Sejong Hospital, Incheon, Republic of Korea

**Keywords:** adipokine, biomarker, cytokine, prediabetes, diabetes, cohort study

## Abstract

**Context:**

The long-term association between multiple cytokines and progression to diabetes is still uncertain.

**Objective:**

To identify which cytokines could predict progression to prediabetes and type 2 diabetes over 10 years.

**Methods:**

The study included 912 participants aged 40 to 69 years at baseline from the Ansung cohort, part of the Korea Genome Epidemiology Study. At baseline, a 75-g oral glucose tolerance test and 8 cytokines were measured: plasminogen activator inhibitor 1 (PAI-1), resistin, interleukin 6, leptin, monocyte chemoattractant protein 1, tumor necrosis factor alpha, retinol binding protein 4 (RBP4), and adiponectin. People with normal glucose tolerance (NGT, n = 241) and prediabetes (n = 330) were followed-up biennially for 10 years. Multinomial logistic regression analysis was used to evaluate the predictability of cytokines on the new-onset prediabetes and type 2 diabetes.

**Results:**

At 10 years, 38 (15.8%) and 82 (34.0%) of those with NGT had converted to prediabetes and type 2 diabetes, respectively. Of those with prediabetes, 228 (69.1%) had converted to type 2 diabetes. In people with NGT or prediabetes at baseline, the highest tertile of RBP4 was associated with a 5.48-fold and 2.43-fold higher risk of progression to type 2 diabetes, respectively. The odds for converting from NGT to prediabetes in the highest tertile of PAI-1 and the lowest tertile of adiponectin were 3.23 and 3.37, respectively. In people with prediabetes at baseline, those in the highest tertile of resistin were 2.94 time more likely to develop type 2 diabetes (all *P* < 0.05).

**Conclusions:**

In this 10-year prospective study, NGT with higher serum RBP4 and PAI-1, and with lower adiponectin were associated with new-onset prediabetes and type 2 diabetes.

The prevalence of type 2 diabetes is increasing dramatically worldwide. According to the *Diabetes Atlas*, a recent report from the International Diabetes Federation, more than 400 million people currently have diabetes, and this will increase to more than 600 million in 2045 (1). At the same time, impaired glucose tolerance (IGT), which increases the risk of developing type 2 diabetes, affects more than 350 million people. Type 2 diabetes is a major cause of cardiovascular disease and overall mortality, and the socioeconomic burden will increase as the prevalence of type 2 diabetes increase. Given the importance of predicting and preventing type 2 diabetes, various efforts have been made to find ways to identify people at high risk of diabetes.

Older age, higher body mass index (BMI), and increased fasting glucose and insulin concentrations are known as predictors of the development of type 2 diabetes in nondiabetic people, as shown in one study in which converters to diabetes had higher values of these traditional factors related insulin resistance than nonconverters (2). Beyond conventional risk factors for type 2 diabetes, inflammatory markers such as fibrinogen, C-reactive protein (CRP), and interleukin-6 (IL-6) are also contributors to the development of type 2 diabetes (3).

Hypertrophy of adipocytes is associated with chronic inflammation and obesity-related type 2 diabetes because adipocytes produce cytokines, which can affect energy metabolism (4, 5). Secreted by adipocytes, adipokines can affect insulin sensitivity and the proinflammatory process (6). Several studies have suggested the clinical roles of adipokines for predicting type 2 diabetes (7, 8). However, little is known about associations between multiple cytokines including adipokines and the development of type 2 diabetes in people with normal glucose tolerance (NGT) and prediabetes over the long-term follow-up.

We conducted a prospective, longitudinal, community-based observational study to measure adipokines and cytokine levels in people without diabetes at the baseline and whether these levels could be used to predict the changes in glucose metabolism over 10 years. We also investigated whether cytokine levels were associated with the conversion to prediabetes or type 2 diabetes after adjustment for known traditional risk factors.

## Materials and Methods

### Study design and participants

The present study was conducted within the Ansung cohort study, a part of the Korean Genome and Epidemiology Study (KoGES), which is an ongoing, prospective, longitudinal, and community-based cohort study to examine trends in diabetes and associated risk factors (9). The detailed design and protocols of the KoGES have been described previously (10). Brieﬂy, this prospective cohort study of 5018 participants aged 40 to 69 years performed the baseline survey in 2001–2002, and biennial follow-up examinations are ongoing. This present study included 912 adults for whom the levels of 8 cytokines were measured at baseline during the period 2001–2002. Participants without baseline glucose status (n = 3), who were diagnosed with type 2 diabetes at baseline (n = 213), or who were not followed-up (n = 125) were excluded. The case subjects were defined as those with NGT at the onset who converted to prediabetes or type 2 diabetes after the 10-year follow-up. These people were selected and then, for each case subject, a control subject who had not developed prediabetes or type 2 diabetes at the 10-year follow-up was selected from the baseline population matched for age (within 10 years), BMI (within 1 kg/m^2^), and sex. Similarly, of the participants with prediabetes at the onset, case subjects who developed incident type 2 diabetes were first selected, and control subjects who did not convert to type 2 diabetes were selected as control subjects matched by age, BMI, and sex. Finally, 241 participants with NGT and 330 with prediabetes at the baseline were enrolled and were regularly examined every 2 years for 10 years.

Anthropometric parameters and blood pressure were measured by standard methods. Fasting plasma glucose (FPG) and insulin levels, lipid profile, CRP level, and the activities of hepatic enzymes were measured in a central laboratory. After an 8- to 14-hour overnight fast, all participants underwent a 2-hour 75-g oral glucose tolerance test (OGTT) at the baseline and then biennially. Hemoglobin A1c (HbA1c) level was measured using high-performance liquid chromatography (Variant II; Bio-Rad Laboratories, Hercules, CA, USA). Insulin resistance and pancreatic β-cell function were calculated using the homeostasis model assessment (HOMA-IR and HOMA-β, respectively).

All of the participants provided written informed consent. The study protocol was approved by the ethics committee of the Korean Center for Disease Control and the Ajou University School of Medicine Institutional Review Board (AJIRB-CRO-07-012).

### Measurement of cytokine levels

The concentrations of 8 cytokines were measured at the baseline using human serum adipokine LINCOplex kits (Linco Research, St Charles, MO). The cytokines were plasminogen activator inhibitor 1 (PAI-1), resistin, IL-6, leptin, monocyte chemoattractant protein-1 (MCP-1), tumor necrosis factor α (TNF-α), retinol-binding protein 4 (RBP4), and adiponectin. The intra-assay variations for the 8 cytokines were 1.4% to 7.9%, and the inter-assay variations were < 21%. The average recovery for the linearity of dilution was 90% to 116%. The antibody pairs used in this assay were specific and no significant cross-reactivity within this panel was observed.

### Statistical analysis

All continuous variables with a normal distribution are expressed as the mean ± standard deviation (SD). Variables with a skewed distribution were logarithmically transformed for statistical analysis. Analysis of variance (ANOVA) with Scheffe post hoc analysis and linear-by-linear association test were used to compare baseline clinical characteristics between the groups according to the outcomes at the 10-year point for the subjects with NGT and prediabetes at baseline, respectively. Pearson correlation analysis was used identify significant correlations between the concentrations of the 8 cytokines and various metabolic parameters. Multinomial logistic regression analysis was used to identify predictors of new-onset prediabetes and type 2 diabetes at the 10-year follow-up. The data were adjusted for traditional risk factors such as age, sex, waist circumference, systolic blood pressure, and the levels of FPG, fasting serum insulin, alanine aminotransferase (ALT), and CRP. The cytokine concentrations were divided into tertiles. In order to investigate the potential diagnostic utility of the specific cytokines for type 2 diabetes or prediabetes, receiver operating characteristic curve analysis was used. Statistical analyses were performed using SPSS software for Windows (version 21.0; IBM Corp., Armonk, NY). A *P* value < 0.05 was considered to be significant.

## Results

### Baseline clinical characteristics of participants with NGT and prediabetes at baseline and comparisons according to the glucose tolerance status at the 10-year follow-up (Tables 1 and 2)

**Table 1.** Baseline Characteristics of Participants With Normal Glucose Tolerance (NGT) According To 10-Year Follow-Up Glucose Tolerance Status

		10-Year Follow-up Glucose Tolerance Status			
Variable	NGT at Baseline (n = 241)	NGT (n = 121)	Prediabetes (n = 38)	Type 2 Diabetes (n = 82)	*P* Value
Age, years	51.5 ± 8.7	48.4 ± 6.7	50.2 ± 8.1	56.8 ± 9.1^† ‡^	<0.001
Male, n (%)	144 (59.8)	71 (58.7)	25 (65.8)	48 (58.5)	0.965
Smoking status (never), n (%)	112 (46.9)	62 (51.7)	16 (43.2)	34 (41.5)	0.156
Alcohol intake (≥60 kcal/day), n (%)	65 (28.3)	34 (29.6)	11 (31.4)	20 (25.0)	0.509
Family history of diabetes, n (%)	32 (13.3)	14 (11.6)	10 (26.3)^†^	8 (9.8)^†^	0.863
Moderate physical activity (≥1 /week), n (%)	59 (24.5)	31 (25.6)	12 (31.6)	16 (19.5)	0.367
Blood pressure. Mm Hg					
Systolic	115 ± 16	110 ± 10	113 ± 10	124 ± 20^† ‡^	<0.001
Diastolic	75 ± 9	72 ± 7	74 ± 7	80 ± 11^† ‡^	<0.001
BMI, kg/m^2^	24.1 ± 3.2	23.3 ± 2.7	25.0 ± 2.9^†^	24.9 ± 3.7^†^	<0.001
Waist circumference, cm	82.6 ± 8.8	79.1 ± 7.0	84.5 ± 7.6^†^	86.7 ± 9.7^†^	<0.001
Percent body fat, %	23.2 ± 7.8	21.4 ± 7.3	23.6 ± 6.6	25.6 ± 8.4^†^	<0.001
HbA1c, %	5.4 ± 0.4	5.2 ± 0.3	5.5 ± 0.3^†^	5.7 ± 0.5^† ‡^	<0.001
Fasting plasma glucose, mmol/L	4.7 ± 0.4	4.6 ± 0.4	4.8 ± 0.4^†^	4.9 ± 0.4^†^	<0.001
2-h plasma glucose, mmol/L	5.8 ± 1.2	5.4 ± 1.1	6.0 ± 1.1^†^	6.2 ± 1.2^†^	<0.001
Fasting insulin, μU/mL	7.9 ± 6.2	7.1 ± 3.3	9.7 ± 11.1	8.3 ± 6.2	0.181
HOMA-IR^*^	1.7 ± 1.3	1.5 ±0.7	2.1 ± 2.3	1.8 ± 1.2	0.050
HOMA-β ^*^, %	148.4 ± 160.3	143.1 ± 82.7	170.9 ± 207.5	145.8 ± 214.5	0.224
Total cholesterol, mg/dL	182.9 ± 32.9	179.8 ± 27.7	177.4 ± 33.0	189.9 ± 38.5	0.031
ALT^*^, IU/L	25.3 ± 16.1	23.5 ± 14.1	23.4 ± 10.7	28.9 ± 20.1^†^	0.011
CRP^*^, mg/dL	0.2 ± 0.6	0.3 ± 0.8	0.2 ± 0.1	0.2 ± 0.2	0.190
Creatinine, mg/dL	0.8 ± 0.2	0.8 ± 0.2	0.8 ± 0.2	0.8 ± 0.2	0.508
PAI-1^*^, ng/mL	38.51 ± 12.39	37.22 ± 12.92	40.38 ± 9.91	39.55 ±12.56	0.141
Resistin^*^, ng/mL	19.88 ± 11.16	18.47 ±10.42	20.03 ±11.89	21.90 ±11.68	0.028
IL-6^*^, pg/mL	2.63 ± 3.95	2.35 ± 4.21	1.77 ± 1.83	3.41 ± 4.26†	0.008
Leptin^*^, ng/mL	7.14 ± 7.53	6.00 ±6.57	6.80 ±7.22	9.00 ±8.64†	0.010
MCP-1^*^, pg/mL	252.9 ± 93.6	241.2 ± 88.6	246.4±91.6	273.2 ± 99.4	0.018
TNF-α ^*^, pg/mL	6.07 ± 3.58	6.07 ± 3.90	6.52 ± 3.71	5.87 ± 3.01	0.884
RBP4^*^, g/mL	135.8 ± 72.7	111.7 ± 54.7	124.7 ± 55.7	176.5 ±84.9^† ‡^	<0.001
Adiponectin^*^, g/mL	3.14 ± 2.96	3.54 ± 2.96	2.03 ± 2.15†	3.06 ± 3.16	0.133

Data are n (%) or mean ± SD. Abbreviations: ALT, alanine aminotransferase; BMI, body mass index; HbA1c, glycated haemoglobin; HOMA-β, homeostatic model assessment of β-cell; HOMA-IR, homeostatic model assessment for insulin resistance; CRP, C-reactive protein; IL-6, interleukin-6; MCP-1, monocyte chemoattractant protein-1; PAI-1, plasminogen activator inhibitor-1; RBP4, retinol binding protein 4; TNF-α, tumour necrosis factor α. The *P* -values were from ANOVA with Scheffe post hoc analysis and linear-by-linear association test. *Analyses were performed after logarithmical transformation; ^†^*P* < 0.05 compared with NGT; ^‡^*P* < 0.05 compared with prediabetes.

**Table 2.** Baseline Characteristics of Participants With Prediabetes According To 10-Year Follow-up Glucose Tolerance Status

		10-Year Follow-up Glucose Tolerance Status			
Variable	Prediabetes at Baseline (n = 330)	NGT (n = 48)	Prediabetes (n = 54)	Type 2 Diabetes (n = 228)	*P* value
Age, years	56.7 ± 8.5	54.6 ± 8.6	56.2 ± 8.5	57.3 ± 8.4	0.051
Male, n (%)	139 (42.1)	15 (31.3)	21 (38.9)	103 (45.2)	0.065
Smoking status (never), n (%)	194 (59.3)	34 (70.8)	36 (66.7)	124 (55.1)	0.016
Alcohol intake (≥60 kcal/day), n (%)	70 (21.9)	9 (19.6)	4 (7.4)	57 (25.9)^‡^	0.067
Family history of diabetes, n (%)	38 (11.5)	2 (4.2)	4 (7.4)	32 (14.0)	0.030
Moderate physical activity (≥1 /week), n (%)	92 (27.9)	17 (35.4)	14 (25.9)	61 (26.8)	0.302
Blood pressure, mm Hg					
Systolic	126 ± 18	119 ± 16	125 ± 19	127 ± 18^†^	0.007
Diastolic	79 ±11	76 ± 9	79 ± 11	80 ± 11	0.023
BMI, kg/m^2^	25.3 ± 3.5	24.2 ± 3.3	25.1 ± 3.3	25.6 ± 3.6^†^	0.011
Waist circumference, cm	87.2 ± 8.7	84.3 ± 9.5	86.1 ± 8.6	88.1 ± 8.5^†^	0.007
Percent body fat, %	28.9 ± 7.5	27.1 ± 7.3	28.6 ± 7.9	29.3 ± 7.4	0.059
HbA1c, %	5.8 ± 0.5	5.3 ± 0.3	5.5 ± 0.3	5.9 ± 0.6^†‡^	<0.001
Fasting plasma glucose, mmol/L	5.1 ± 0.6	4.9 ± 0.5	4.9 ± 0.6^‡^	5.2 ± 0.7^†‡^	0.005
2-h plasma glucose, mmol/L	9.0 ± 1.1	8.6 ± 0.9	8.6 ± 1.1^‡^	9.2 ± 1.2^‡^	0.002
Fasting insulin, μU/mL	8.8 ± 5.2	7.9 ± 3.5	8.2 ± 4.5	9.2 ± 5.6^‡^	0.069
HOMA-IR^*^	2.0 ± 1.3	1.8 ± 0.9	1.8 ± 1.0	2.1 ± 1.4^†‡^	0.024
HOMA-β ^*^, %	141.9 ± 271.2	123.0 ± 59.8	132.0 ± 90.8	148.2 ± 322.2	0.967
Total cholesterol, mg/dL	185.1 ± 100.6	163.4 ± 117.5	184.7 ± 104.4	189.8 ± 95.6^†^	0.009
ALT^*^, IU/L	29.0 ± 37.0	23.8 ± 20.7	24.4 ± 11.2	31.2 ± 43.0^†^	0.005
CRP^*^, mg/dL	0.3 ± 0.9	0.2 ± 0.3	0.3 ± 0.3	0.4 ± 1.1	0.177
Creatinine, mg/dL	0.8 ± 0.2	0.8 ± 0.2	0.8 ± 0.2	0.8 ± 0.2	0.844
PAI-1^*^, ng/mL	40.16 ± 11.42	39.09 ± 11.06	39.99 ± 10.51	40.43 ± 11.74	0.535
Resistin^*^, ng/mL	24.65±43.69	16.74 ± 9.68	19.46 ± 16.24	27.54 ± 51.55^†^	0.003
IL-6^*^, pg/mL	4.9 ± 8.2	4.3 ± 5.3	5.8 ± 5.3	4.8 ± 9.1^‡^	0.683
Leptin^*^, ng/mL	13.11 ± 11.85	11.95 ± 11.08	12.83 ± 12.48	13.42 ± 11.89	0.749
MCP-1^*^, pg/mL	277.8 ± 91.9	275.7 ± 82.3	279.4 ± 89.1	277.8 ± 94.8	0.945
TNF-α ^*^, pg/mL	7.4 ± 5.3	7.8 ± 3.0	8.8 ± 6.6	6.9 ± 5.3	0.020
RBP4^*^, g/mL	160.7 ± 81.3	143.6 ± 71.2	137.3 ± 77.1	169.9±82.8^‡^	0.033
Adiponectin^*^, g/mL	2.98 ± 2.73	3.63 ± 3.48	3.22 ± 2.77	2.79 ± 2.53	0.072

Data are n (%) or mean ± SD. Abbreviations: ALT, alanine aminotransferase; BMI, body mass index; HbA1c, glycated haemoglobin; HOMA-β, homeostatic model assessment of β-cell; HOMA-IR, homeostatic model assessment for insulin resistance; CRP, C-reactive protein; IL-6, interleukin-6; MCP-1, monocyte chemoattractant protein-1; PAI-1, plasminogen activator inhibitor-1; RBP4, retinol binding protein 4; TNF-α, tumour necrosis factor α. The *P* -values were from ANOVA with Scheffe post hoc analysis and linear-by-linear association test. *Analyses were performed after logarithmic transformation; ^†^*P* < 0.05 compared with NGT; ^‡^*P* < 0.05 compared with prediabetes.

Table 1 shows the baseline clinical characteristics of participants with NGT and comparisons of the 3 groups according to their glucose tolerance status at the 10-year follow-up. Among 241 participants who had NGT at baseline, 120 developed prediabetes, defined as impaired fasting glucose (IFG) or IGT (n = 38) or type 2 diabetes (n = 82) at 10-year follow-up examination. The ratios of men and women did not differ between the 3 groups. Participants who converted to type 2 diabetes were older than other groups. A higher percentage of those who converted to prediabetes had a familial history of diabetes compared with the other groups. Baseline waist circumference and BMI were greater in those who converted to prediabetes or type 2 diabetes compared with nonconverters. Those who developed type 2 diabetes had the highest baseline body fat percentage. Of the biochemical parameters, the levels of FPG, postprandial 2-hour plasma glucose (PP2), HbA1c, and ALT at the baseline were significantly higher and tended to increase in those who converted to prediabetes or type 2 diabetes. Baseline high-density lipoprotein cholesterol level was lower in type 2 diabetes converters than in nonconverters (data not shown)

In the NGT group at the baseline, resistin level tended to increase according to glucose tolerance status at the 10-year follow-up. IL-6, leptin, and RBP4 levels were higher in those who developed type 2 diabetes than in the other groups. The levels of these cytokines tended to increase according to progression of glycemic status at the 10-year follow-up assessment. Adiponectin level was lower in those who developed prediabetes than in nonconverters.

Table 2 shows the baseline clinical characteristics in the group with prediabetes and comparisons of the 3 groups according to their glucose tolerance results after the 10-year follow-up evaluation. At the baseline, 330 participants had IFG or IGT. At 10 years, 228 with prediabetes had developed type 2 diabetes. There were no differences in mean age, sex distribution, physical activity level, and alcohol consumption between the 3 groups. Baseline systolic blood pressure, waist circumference, and BMI were significantly higher in participants who developed type 2 diabetes. Of the biochemical parameters, the levels of FPG, PP2, HbA1c, and ALT were significantly higher in type 2 diabetes converters than in the other groups

In the prediabetes group at the baseline, resistin level was significantly higher in type 2 diabetes converters compared with other groups. RBP4 level was significantly higher in the prediabetes-to–type 2 diabetes group than in those who were remained in the prediabetes group. Resistin and RBP4 levels tended to increase with progression of glycemic status at 10 years.

Detailed results of the 75-g OGTT conducted at each of the 2-year intervals can be found in Reference 11.

### Associations between the levels of the 8 cytokines and metabolic risk factors at baseline

As shown in Table 3, age correlated positively with multiple cytokines except for PAI-1 and leptin. Obesity indices such as waist circumference and percent body fat correlated significantly positively with serum leptin level. Serum levels of PAI-1, leptin, TNF-α, and IL-6 were weakly but significantly associated with systolic blood pressure, as well as with levels of FPG, PP2, and total cholesterol. Serum RBP4 and resistin levels correlated with HbA1c and PP2 levels, respectively. Serum adiponectin level correlated inversely with waist circumference and levels of FPG and ALT. Fasting serum insulin level and HOMA-IR were strongly positively associated with leptin level and inversely with adiponectin level. PAI-1 level correlated significantly with ALT and CRP levels. CRP level also correlated positively with resistin, leptin, and MCP-1 levels.

**Table 3.** Correlations Between Baseline Characteristics and the Levels Of 8 Cytokines

Variable	PAI-1	RBP4	Resistin	Leptin	TNF-α	MCP1	IL-6	Adiponectin
Age, years	−0.036	0.116^‡^	0.206^‡^	0.018	0.216^‡^	0.088^†^	0.131^‡^	0.143^‡^
Waist circumference, cm	0.171^‡^	0.121^‡^	0.009	0.533^‡^	0.084^†^	0.098^†^	0.163^‡^	−0.205^‡^
Percent body fat, %	0.008	0.084^†^	−0.007	0.778^‡^	0.015	−0.022	0.017	0.084
SBP, mmHg	0.159^‡^	0.080	0.078	0.162^‡^	0.084^†^	−0.058	0.211^‡^	−0.024
HbA1c, %	0.114^‡^	0.116^‡^	0.065	0.256^‡^	0.057	0.010	0.214^‡^	−0.051
Fasting plasma glucose, mg/dL	0.135^‡^	−0.019	0.049	0.160^‡^	0.114^‡^	0.056	0.173^‡^	−0.195^‡^
2-h plasma glucose, mg/dL	0.101^†^	0.077	0.172^‡^	0.365^‡^	0.116^‡^	0.090^†^	0.165^‡^	−0.060
Fasting serum insulin^*^, μU/mL	0.065	0.031	−0.069	0.424^‡^	0.066	0.013	0.012	−0.109^†^
HOMA-IR^*^	0.087	0.026	−0.058	0.433^‡^	0.085†	0.024	0.043	−0.140^‡^
HOMA-β ^*^, %	−0.023	0.043	−0.091^†^	0.235^‡^	−0.035	−0.057	−0.098^†^	0.043
Total cholesterol, mg/dL	0.156^‡^	0.005	0.010	0.287^‡^	0.184^‡^	0.070	0.224^‡^	−0.020
ALT^*^, IU/L	0.190^‡^	0.014	−0.003	−0.061	0.078	0.028	0.183^‡^	−0.211^‡^
CRP^*^, mg/dL	0.150^‡^	0.065	0.222^‡^	0.124^‡^	0.064	0.136^‡^	0.064	−0.068

Abbreviations: ALT, alanine aminotransferase; HbA1c, glycated haemoglobin; HOMA-β, homeostatic model assessment of β-cell; HOMA-IR, homeostatic model assessment for insulin resistance; CRP, C-reactive protein; SBP, systolic blood pressure. Pearson’s correlation coefficients (*r*) are given. *Analyses were performed after logarithmic transformation; ^†^*P* < 0.05; ^‡^*P* < 0.01.

### **Multinomial logistic regression analysis to identify predictors of the progression to prediabetes or type 2 diabetes after 10 years based on baseline NGT and prediabetes (** Fig. 1)

**Figure 1.**
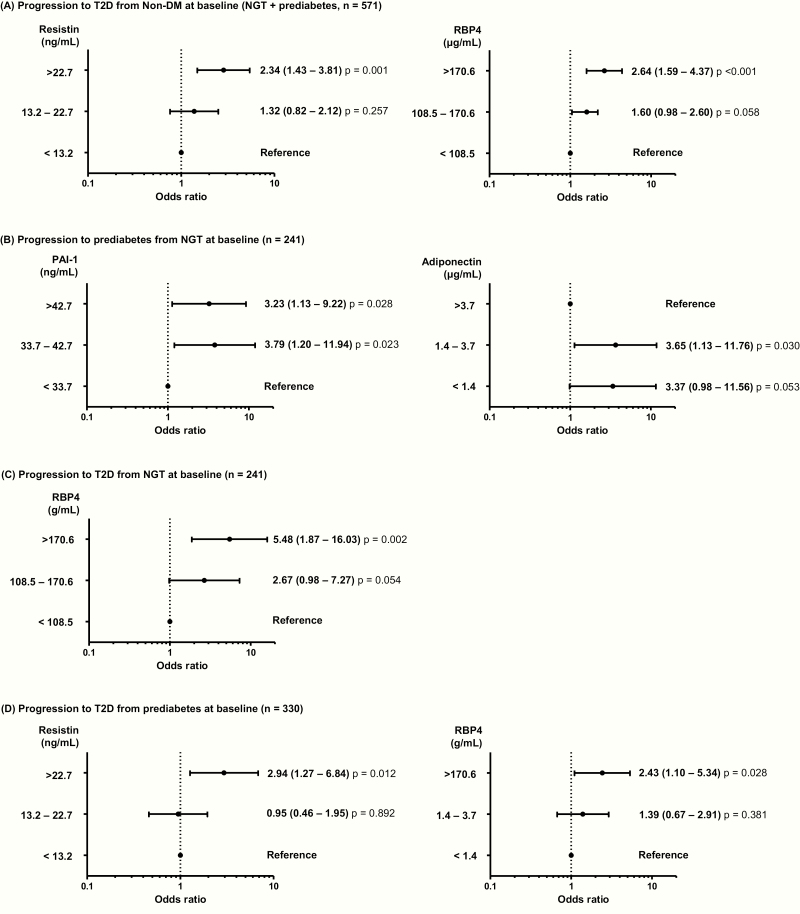
Progression to prediabetes and type 2 diabetes. (A) Risk factors for the progression to type 2 diabetes at the 10-year follow-up examination in participants with normal glucose tolerance (NGT) or prediabetes (n = 571). (B) Risk factors for the progression to prediabetes at the 10-year follow-up examination in participants with NGT (n = 241). (C) Risk factors for the progression to type 2 diabetes at the 10-year follow-up examination in participants with NGT (n = 241). *D*: Risk factors for the progression to type 2 diabetes at the 10-year follow-up examination in participants with prediabetes (n = 330). Multinomial logistic regression model was used after adjustment for age, sex, waist circumference, systolic blood pressure, and concentrations of fasting plasma glucose, alanine aminotransferase, and C-reactive protein. The levels of cytokines are expressed as tertiles, and odds ratios (95% CIs) are shown above the black circle.

Participants with the highest tertile of resistin and RBP4 were 2.34- and 2.64-times more likely to develop type 2 diabetes compared with those in the lowest tertile (95% conﬁdence interval [CI], 1.43–3.81; *P* = 0.001; and 95% CI, 1.59–4.37; *P* < 0.001, respectively) (Fig. 1A). Participants in the NGT group with the highest tertile of PAI-1 were 3.23-times more likely to develop prediabetes compared with those in the lowest tertile (95% CI, 1.13–9.22; *P* = 0.028) (Fig. 1B). In the NGT group, those in the middle and lowest tertiles of adiponectin level were 3.65- and 3.37-times more likely to develop prediabetes compared with those in the highest tertile of adiponectin (95% CI, 1.13–11.76; *P* = 0.030; and 95% CI, 0.98–11.58; *P* = 0.053, respectively). Participants in the NGT group in the highest RBP4 tertile were 5.48-times more likely to develop type 2 diabetes than were those in the lowest tertile (95% CI, 1.87–16.03; *P* = 0.002) (Fig. 1C).

In the prediabetes group, participants in the highest tertile of resistin were 2.94-times more likely to develop type 2 diabetes more compared with those in the lowest tertile (95% CI, 1.27–6.84; *P* = 0.012) (Fig. 1D). Those with prediabetes in the highest tertile of RBP4 was 2.43-times more likely to convert to type 2 diabetes than were those in the lowest tertile (95% CI, 1.10–5.34; *P* = 0.028).

The ROC analysis of the 3 significantly increased cytokines (RBP4, PAI-1, resistin) and the 1 significantly decreased cytokine (adiponectin) can be found in Reference 11.

## Discussion

In this study, we found that serum PAI-1, adiponectin, resistin, and RBP4 levels were significant biomarkers for predicting the future progression to prediabetes or type 2 diabetes after adjusting for parameters related to inflammation and insulin resistance. In people without diabetes at the baseline, resistin and RBP4 levels were significant predictors of type 2 diabetes development at 10 years.

RBP4 is the retinol carrier in the blood. Yang et al revealed its role as an adipokine related to type 2 diabetes in 2005 (13). They demonstrated that serum RBP4 level was higher in humans with obesity and type 2 diabetes as well as in insulin-resistant mice. This association between elevated serum RBP4 level and insulin resistance has also been reported in nonobese, nondiabetic people with a strong familial history of type 2 diabetes. Our group has also reported this association in women with previous gestational diabetes (13) and in people with obesity, IGT, or type 2 diabetes (14). However, some reports do not support an association between RBP4 level and development of type 2 diabetes. Some studies have reported no significant correlation between RBP4 level and insulin resistance and that RBP4 level is not helpful for predicting future diabetes (15, 16). In this present study, among participants without diabetes at the baseline (NGT and prediabetes), baseline RBP4 level was significantly higher in type 2 diabetes converters than nonconverters at the 10-year follow-up. After adjustment for traditional risk factors, RBP4 level was a clear predictor of future diabetes.

Resistin level also correlated strongly with progression to type 2 diabetes in our study. Resistin is produced mainly by white adipose tissue (17). Despite associations between resistin and obesity or insulin resistance in animal models, its role in human disease is not clear. Some studies have suggested that resistin might not play a main role in insulin resistance or energy metabolism in humans (18, 19). However, another study showed that resistin expression was greater in pancreatic islet cells of type 2 diabetes patients than in a control group (20). Similarly, we also found that serum resistin level was significantly associated with PP2 level and that participants in the highest tertile of baseline resistin had a > 2-fold increased risk of progression to type 2 diabetes than did those in the lowest tertile.

Negative correlations between adiponectin level and obesity, insulin resistance, or atherosclerosis have been reported (7, 21). Previous studies have suggested that a lower adiponectin level is a predictor of the future development of diabetes (7, 22, 23). In this study, we also found significant inverse correlations between serum adiponectin level and waist circumference, HOMA-IR, triglyceride level, and ALT. A lower adiponectin level was an independent risk factor for progression to prediabetes from NGT after correction for other risk factors.

PAI-1 is a prothrombotic cytokine secreted by various cells, including adipocytes (24). Previous studies have reported a stronger association between PAI-1 level and cardiovascular disease than between PAI-1 and obesity, insulin resistance, or type 2 diabetes (25, 26). Studies have focused on the role of PAI-1 in human adipose tissue and how an elevated PAI-1 level contributes to central obesity (27), metabolic syndrome (28), and future IGT or type 2 diabetes (29). We also found that increased serum PAI-1 level was a significant independent predictor of the progression from NGT to prediabetes after adjustment of confounding variables. A recent study reported that PAI-1 level was higher in people with high HbA1c level, IFG, or IGT than in those with NGT or diabetes (30). These results suggest that people with prediabetes may have a higher risk of type 2 diabetes as well as cardiovascular disease.

A meta-analysis reported that increased levels of inflammatory markers such as IL-6 and CRP significantly increase the risk of incident type 2 diabetes (3). Elevated baseline leptin level is associated with the future development of type 2 diabetes (8). Similarly, we found that baseline IL-6 and leptin levels were higher in participants with glycemic progression at the 10-year follow-up and that leptin level was significantly associated with higher glucose level, insulin resistance, and dyslipidemia. However, these relationships were not significant after adjustment for confounding factors such as BMI and levels of FPG and fasting serum insulin. MCP-1 and TNF-α are proinflammatory cytokines secreted by adipose tissue (31). In our study, TNF-α level was associated with hyperglycemia, insulin resistance, and dyslipidemia, and MCP-1 level was associated with CRP level, but TNF-α and MCP-1 levels were not independent contributors to diabetes progression after adjustment for metabolic parameters.

This present study has several strengths. The primary strength is that the data are based on a large community-based longitudinal cohort with a homogeneous ethnic background who were followed for more than 10 years. Second, we used a stringent method such as the 75-g OGTT in a central laboratory to identify prediabetes and type 2 diabetes. Third, the levels of multiple adipokines, including RBP4 and resistin, were measured, although their roles in humans are controversial. Our study has some limitations. All participants were enrolled as middle-aged adults in a rural area of Korea, and additional studies of people at various ages and races are needed. We did not repeat the measurement of cytokines level, and we could not study the potential effects of changes of cytokines level. We performed multiple comparison analysis with adjustment of possible confounding factors, but as a limitation, type 1 errors cannot be completely avoided in this observational study.

In conclusion, during the 10-year observation period, higher levels of serum RBP4, resistin, and PAI-1 were associated with the risk of developing prediabetes or type 2 diabetes and lower serum adiponectin level was a predictor of future prediabetes. We suggest that measuring the levels of various adipokines and cytokines in healthy adults without diabetes may be helpful for predicting the risk of future diabetes.

## Abbreviations

ALT: alanine aminotransferase
BMI: body mass index
CRP: C-reactive protein
FPG: fasting plasma glucose
HbA1c: hemoglobin A1c
HOMA2-β: homeostasis model assessment for β-cell function
HOMA-IR: homeostasis model assessment for insulin resistance
IFG: impaired fasting glucose
IGT: impaired glucose tolerance
IL-6: interleukin 6
MCP-1: monocyte chemoattractant protein-1
NGT: normal glucose tolerance
OGTT: oral glucose tolerance test
PP2: postprandial 2-hour plasma glucose
RBP4: retinol-binding protein 4
TNF-α: tumor necrosis factor-α

## Additional Information

***Disclosure Summary:*** The authors have no conflict of interest to disclose.

***Data Availability:*** The datasets generated during and/or analysed during the current study are not publicly available but are available from the corresponding author on reasonable request.

## References

[1] Cho NH, Shaw JE, Karuranga S, et al IDF Diabetes Atlas: Global estimates of diabetes prevalence for 2017 and projections for 2045. Diabetes Res Clin Pract. 2018;138:271–281. 29496507 10.1016/j.diabres.2018.02.023

[2] Haffner SM, Stern MP, Mitchell BD, Hazuda HP, Patterson JK Incidence of type II diabetes in Mexican Americans predicted by fasting insulin and glucose levels, obesity, and body-fat distribution. Diabetes. 1990;39(3):283–288. 2407581 10.2337/diab.39.3.283

[3] Wang X, Bao W, Liu J, et al Inflammatory markers and risk of type 2 diabetes: a systematic review and meta-analysis. Diabetes Care. 2013;36(1):166–175. 23264288 10.2337/dc12-0702 PMC3526249

[4] Kloting N and Bluher M Adipocyte dysfunction, inflammation and metabolic syndrome. Rev Endocr Metab Disord 2014;15:277–287. 25344447 10.1007/s11154-014-9301-0

[5] Catalan V, Gomez-Ambrosi J, Rodriguez A, Salvador J and Fruhbeck G Adipokines in the treatment of diabetes mellitus and obesity. Expert Opin Pharmacother 2009;10:239–254. 19236196 10.1517/14656560802618811

[6] Blüher M Adipokines - removing road blocks to obesity and diabetes therapy. Mol Metab. 2014;3(3):230–240. 24749053 10.1016/j.molmet.2014.01.005 PMC3986498

[7] Snehalatha C, Mukesh B, Simon M, Viswanathan V, Haffner SM, Ramachandran A Plasma adiponectin is an independent predictor of type 2 diabetes in Asian indians. Diabetes Care. 2003;26(12):3226–3229. 14633806 10.2337/diacare.26.12.3226

[8] McNeely MJ, Boyko EJ, Weigle DS, Shofer JB, Chessler SD, Leonnetti DL, Fujimoto WY Association between baseline plasma leptin levels and subsequent development of diabetes in Japanese Americans. Diabetes Care. 1999;22(1):65–70. 10333905 10.2337/diacare.22.1.65

[9] Cho NH, Jang HC, Choi SH, Kim HR, Lee HK, Chan JC, Lim S Abnormal liver function test predicts type 2 diabetes: a community-based prospective study. Diabetes Care. 2007;30(10):2566–2568. 17626893 10.2337/dc07-0106

[10] Shin C, Abbott RD, Lee H, Kim J, Kimm K Prevalence and correlates of orthostatic hypotension in middle-aged men and women in Korea: the Korean Health and Genome Study. J Hum Hypertens. 2004;18(10):717–723. 15116144 10.1038/sj.jhh.1001732

[11] Ku EJ and Choi SH Data from: Estimated association between cytokines and the progression to diabetes: 10-year follow-up from a community-based cohort – supplementary appendix. Figshare Digital Repository 2019. Deposited October 2, 2019. www.doi.org/10.6084/m9.figshare.9928481.v2

[12] Yang Q, Graham TE, Mody N, et al Serum retinol binding protein 4 contributes to insulin resistance in obesity and type 2 diabetes. Nature. 2005;436(7049):356–362. 16034410 10.1038/nature03711

[13] Choi SH, Kwak SH, Youn BS, et al High plasma retinol binding protein-4 and low plasma adiponectin concentrations are associated with severity of glucose intolerance in women with previous gestational diabetes mellitus. J Clin Endocrinol Metab. 2008;93(8):3142–3148. 18492757 10.1210/jc.2007-1755

[14] Graham TE, Yang Q, Blüher M, et al Retinol-binding protein 4 and insulin resistance in lean, obese, and diabetic subjects. N Engl J Med. 2006;354(24):2552–2563. 16775236 10.1056/NEJMoa054862

[15] Rhee EJ, Seo MH, Jeon WS, et al The association of baseline adipocytokine levels with glycemic progression in nondiabetic Korean adults in 4 years of follow-up. Diabetes Res Clin Pract. 2012;98(3):501–507. 23068962 10.1016/j.diabres.2012.09.022

[16] Kaess BM, Enserro DM, McManus DD, et al Cardiometabolic correlates and heritability of fetuin-A, retinol-binding protein 4, and fatty-acid binding protein 4 in the Framingham Heart Study. J Clin Endocrinol Metab. 2012;97(10):E1943–E1947. 22855337 10.1210/jc.2012-1458 PMC3674297

[17] Cantley J The control of insulin secretion by adipokines: current evidence for adipocyte-beta cell endocrine signalling in metabolic homeostasis. Mamm Genome. 2014;25(9-10):442–454. 25146550 10.1007/s00335-014-9538-7

[18] Qi Q, Wang J, Li H, et al Associations of resistin with inflammatory and fibrinolytic markers, insulin resistance, and metabolic syndrome in middle-aged and older Chinese. Eur J Endocrinol. 2008;159(5):585–593. 18753313 10.1530/EJE-08-0427

[19] Lee JH, Chan JL, Yiannakouris N, et al Circulating resistin levels are not associated with obesity or insulin resistance in humans and are not regulated by fasting or leptin administration: cross-sectional and interventional studies in normal, insulin-resistant, and diabetic subjects. J Clin Endocrinol Metab. 2003;88(10):4848–4856. 14557464 10.1210/jc.2003-030519

[20] Al-Salam S, Rashed H, Adeghate E Diabetes mellitus is associated with an increased expression of resistin in human pancreatic islet cells. Islets. 2011;3(5):246–249. 21750415 10.4161/isl.3.5.16427

[21] Ouchi N, Shibata R, Walsh K Cardioprotection by adiponectin. Trends Cardiovasc Med. 2006;16(5):141–146. 16781946 10.1016/j.tcm.2006.03.001 PMC2749293

[22] Choi KM, Lee J, Lee KW, et al Serum adiponectin concentrations predict the developments of type 2 diabetes and the metabolic syndrome in elderly Koreans. Clin Endocrinol (Oxf). 2004;61(1):75–80. 15212647 10.1111/j.1365-2265.2004.02063.x

[23] Daimon M, Oizumi T, Saitoh T, et al; Funagata study Decreased serum levels of adiponectin are a risk factor for the progression to type 2 diabetes in the Japanese Population: the Funagata study. Diabetes Care. 2003;26(7):2015–2020. 12832305 10.2337/diacare.26.7.2015

[24] Samad F, Loskutoff DJ Tissue distribution and regulation of plasminogen activator inhibitor-1 in obese mice. Mol Med. 1996;2(5):568–582. 8898373 PMC2230189

[25] Juhan-Vague I, Alessi MC Plasminogen activator inhibitor 1 and atherothrombosis. Thromb Haemost. 1993;70(1):138–143. 8236090

[26] Hamsten A, Wiman B, de Faire U, Blombäck M Increased plasma levels of a rapid inhibitor of tissue plasminogen activator in young survivors of myocardial infarction. N Engl J Med. 1985;313(25):1557–1563. 3934538 10.1056/NEJM198512193132501

[27] Alessi MC, Peiretti F, Morange P, Henry M, Nalbone G, Juhan-Vague I Production of plasminogen activator inhibitor 1 by human adipose tissue: possible link between visceral fat accumulation and vascular disease. Diabetes. 1997;46(5):860–867. 9133556 10.2337/diab.46.5.860

[28] Ahirwar AK, Jain A, Goswami B, Bhatnagar MK, Bhatacharjee J Imbalance between protective (adiponectin) and prothrombotic (Plasminogen Activator Inhibitor-1) adipokines in metabolic syndrome. Diabetes Metab Syndr. 2014;8(3):152–155. 25042166 10.1016/j.dsx.2014.04.035

[29] Festa A, D’Agostino R Jr, Mykkänen L, Tracy RP, Zaccaro DJ, Hales CN, Haffner SM Relative contribution of insulin and its precursors to fibrinogen and PAI-1 in a large population with different states of glucose tolerance. The Insulin Resistance Atherosclerosis Study (IRAS). Arterioscler Thromb Vasc Biol. 1999;19(3):562–568. 10073958 10.1161/01.atv.19.3.562

[30] Xu L, Jiang CQ, Lam TH, Bao B, Cheng KK, Thomas GN Plasminogen activator inhibitor-1 and HbA1c defined prediabetes: the Guangzhou Biobank Cohort Study-CVD. Clin Endocrinol (Oxf). 2011;74(4):528–531. 21128994 10.1111/j.1365-2265.2010.03948.x

[31] Shoelson SE, Lee J, Goldfine AB Inflammation and insulin resistance. J Clin Invest. 2006;116(7):1793–1801. 16823477 10.1172/JCI29069 PMC1483173

