# CORRIGENDUM FOR “Estimated Association Between Cytokines and the Progression to Diabetes: 10-year Follow-up From a Community-Based Cohort”

**DOI:** 10.1210/clinem/dgaa094

**Published:** 2020-03-14

In the above-named article by Cho NH, Ku EJ, Jung KY, Oh TJ, Kwak SH, Moon JH, Park KS, Jang HC, Kim YJ, and Choi SH (*J Clin Endocrinol Metab.* 2020;105(3):e381–e389; doi: 10.1210/clinem/dgz171), the author’s name Nam Han Cho should instead appear as Nam H. Cho.

